# Hand rejuvenation with fat grafting: A 12-year single-surgeon experience

**DOI:** 10.1007/s00238-017-1337-4

**Published:** 2017-07-18

**Authors:** Fabio Fantozzi

**Affiliations:** Administration, ACEIP (European Surgical Association of Prof. Ivo Pitanguy Alumni), Rome, Italy

**Keywords:** Fat grafting, Hand lipofilling, Hand reshaping, Hand rejuvenation

## Abstract

**Background:**

Fat grafting has been successfully used for reconstructive and esthetic surgery of the breast, face, and other body parts. In this article, we present our protocol for hand fat grafting and over a decade of clinical experience.

**Methods:**

Fat tissue is obtained from the flanks, peri-umbilical region, or internal side of the thigh or knee. No centrifuge machine is used to prevent fat damage. After decantation, fat is injected into the dorsum of the hand using a cannula from the wrist and not from the fingers. Fat is distributed gently above the dorsal deep fascia to avoid perforation of the vessels.

**Results:**

The proposed technique was applied to 65 patients. The amount of fat injected ranged from 10 to 30 cm^3^. No allergic reactions were noticed. Each patient’s progress was followed-up for a minimum of 12 months. Over this period, contour changes and the effects of the procedure(s) on the skin were analyzed. Fifty-six patients (84%) were satisfied with the results during the observation period, 7 patients (12%) were somewhat satisfied and needed one more fat grafting procedure to achieve complete satisfaction, and 2 patients (4%) were dissatisfied with the results. Three cases of temporary swelling of the hands resolved naturally. No long-term complications were seen.

**Conclusions:**

This study covers over a decade of practical experience in applying fat grafts to hands. The procedure is effective in reshaping and rejuvenating the hand as it shows long-lasting results after 1-year follow-up.

## Introduction

**Fig. 1:**
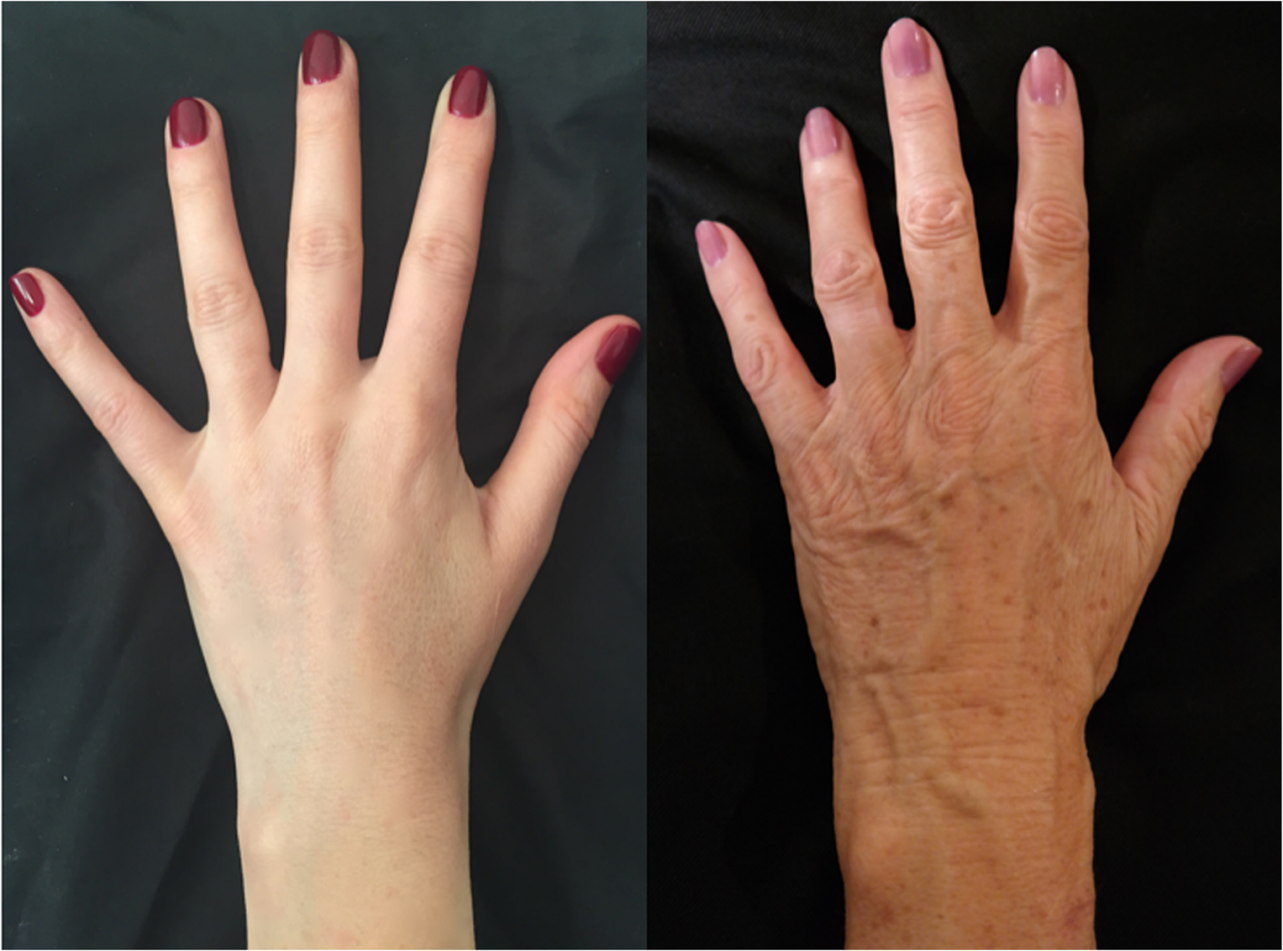
Aesthetic aspects of the elderly hand:deterioration of skin quality, presence of skin flaccidity, abscense of subcutaneous fat tissue, alteration of cutaneous pigmentation and visible veins and tendons on the dorso.

Through history, humans have always been fascinated by the beauty of hands. Hands have been considered a key element characterizing the privileged status of man as well as an immediate means of expressing man’s creativity. In art, hand movements are treated as a real, meaningful language which has been codified, albeit with noticeable variations, over the centuries. In modern medical science, fat grafting is a procedure that has been used in esthetic and reconstructive surgery for many years with good results [1, 2]. Many patients are interested in improving the esthetic appearance of their hands. By using fat grafting, we can provide a better contour in the prominent aged anatomy of the hand and rejuvenate the skin after lipofilling [3]. In order to better analyze hand rejuvenation effects by fat grafting, main characteristics of the hand during different stages of life are summarized in Fig. 1.

The aesthetic  aspects of an elderly hand include deterioration of skin quality with alteration of cutaneous pigmentation, presence of skin flaccidity, visible veins and tendons on the dorso, and absence of subcutaneous fat tissue. Fat grafting can be used to produce two effects: fill and rejuvenate the hand. By using autologous fat obtained, it is possible fill the dorsum of the hand. This type of lipofilling enables visible veins and tendons to be covered, resulting in a hand with uniform contours, like a young hand. Furthermore, the large amount of added fat cells has a beneficial effect on deep and superficial skin tissue, thereby rejuvenating the hand [3].

Adipose tissue transfer is generally performed for treating esthetic problems and deformities of the hand. Most patients undergo treatment only for cosmetic problems; however, some patients require treatment for necessary reconstruction after suffering trauma [3, 4].

The aim of this article was to present our experience and protocol for hand fat grafting to reshape and rejuvenate the hands.

## Material and methods

Between January 2003 and January 2016, a retrospective study of all patients undergoing fat grafting in the dorsum of the hand was conducted. In the analysis, we considered factors such as age, sex, operative time, type of procedure, and complications.

### Surgical technique

**Fig. 2:**
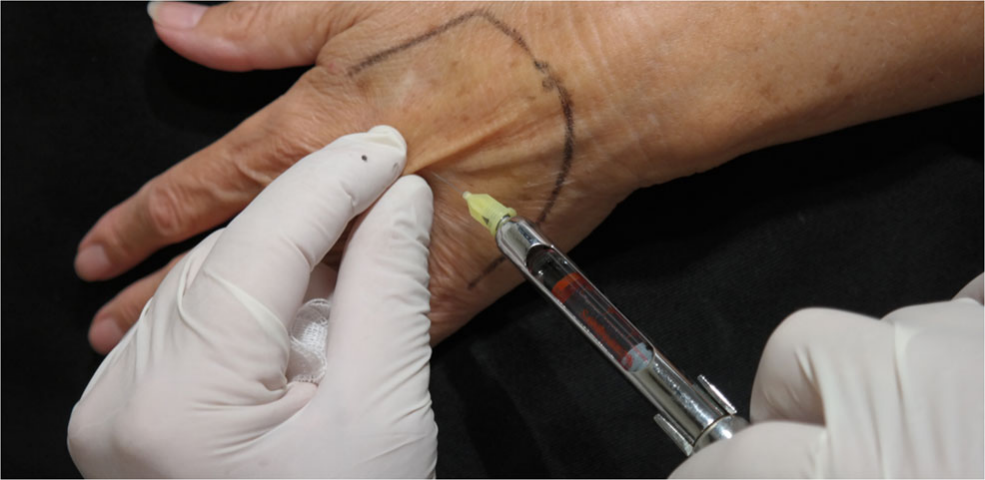
For anaesthetization, 5 mL of the anesthesia solution is injected into the dorsum of the hand

**Fig. 3:**
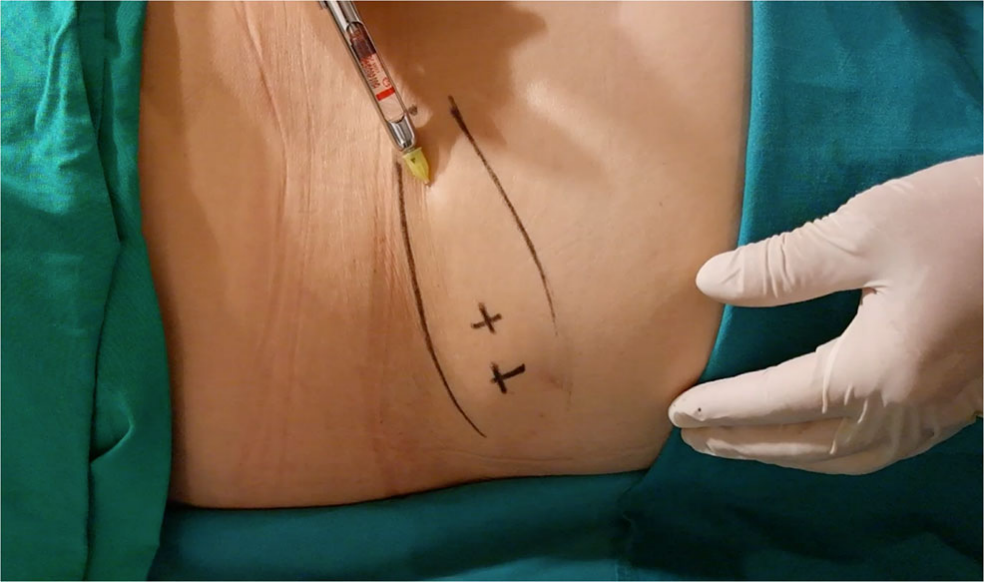
Anesthetic infiltration of the region from where fat tissue is extracted

**Fig. 4:**
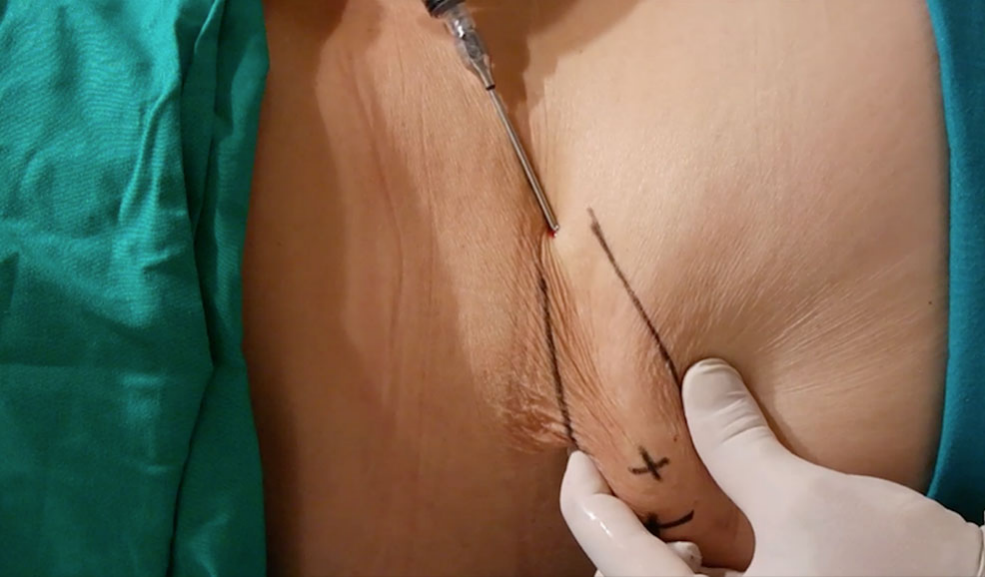
A minimum of 30 cm^3^ of fat tissue is collected

**Fig. 5:**
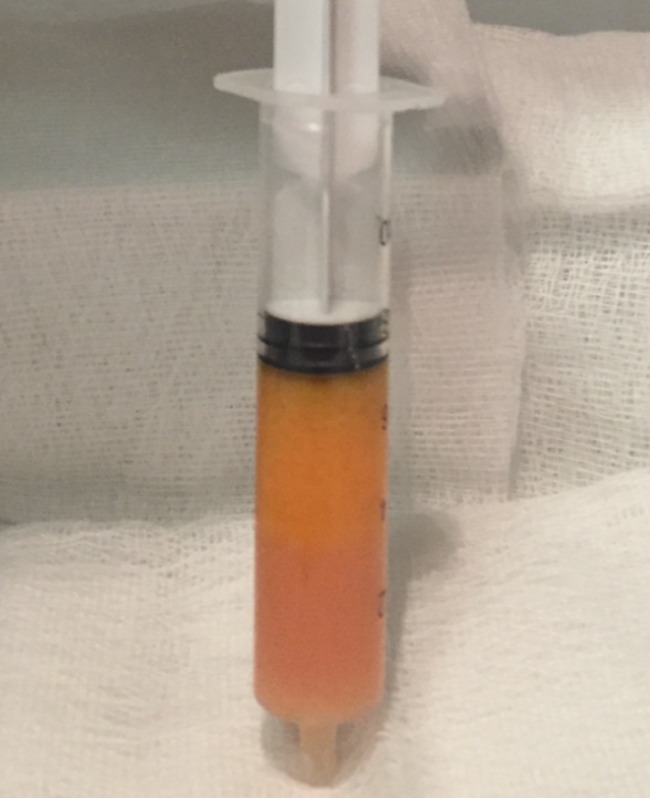
The fat is decanted to allow separation from contaminants such as blood, serum, and oil

**Fig. 6:**
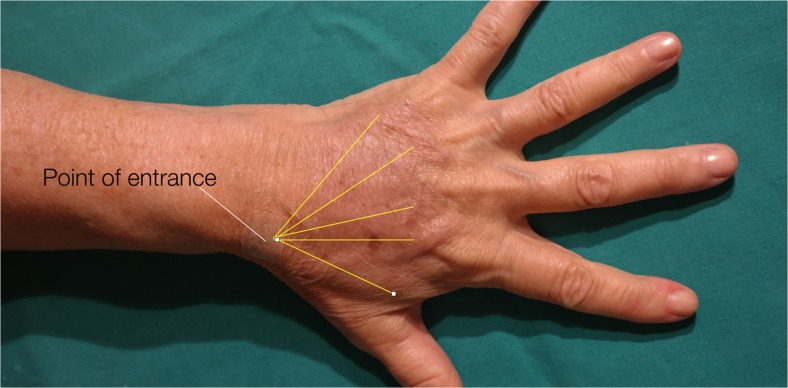
Point of entrance and lines of filling: fat is injected into the dorsum of the hand using a cannula from the wrist

**Fig. 7:**
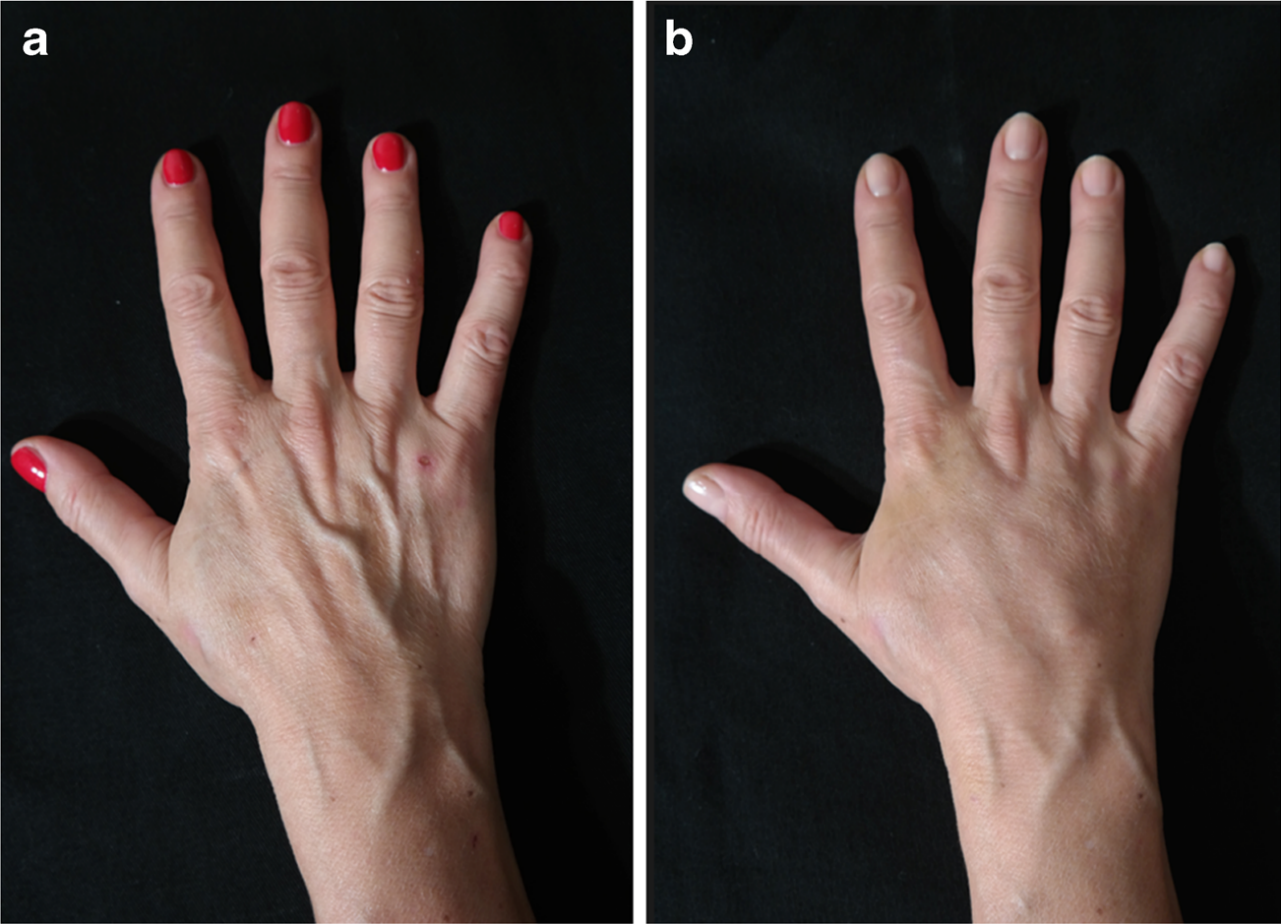
A 41-year-old female patient (right hand): **a** preoperative and **b** postoperative view at 9 months

The area of the dorsum of the hand where fat tissue will be injected must first be defined. The flanks, peri-umbilical region, or internal face of the thigh or knee are usually chosen as the donor areas for extracting fat tissue. Fat grafting into the hands is a relatively simple procedure. It usually requires only local anesthesia and is performed on an ambulatory basis. A combination of naropine (10 mL), adrenaline (1:200.000), and sodium chloride (9 mg/mL and 50 mL) is used as the local anesthesia solution. For anesthetization, 5 mL of the anesthesia solution is injected into the dorsum of the hand (Fig. 2) and 50 mL, into the region from where fat tissue is extracted (Fig. 3). Before starting fat extraction, it is important to wait for a minimum of 20 min to allow for the adrenaline action. Fat extraction requires only ~5 min and it is performed by using a cannula (outer diameter 3.0 mm). A minimum of 30 cm^3^ of fat tissue is collected (Fig. 4). The fat is then decanted for 10 min; contaminants such as blood, serum, and oil are removed; and in this way, the fat is ready to be injected (Fig. 5). Finally, 10 to 30 cm^3^ of fat is injected into the dorsum of the hand. Fat grafting is performed using a cannula (outer diameter 1.4 mm) from the wrist and not from the fingers (Fig. 6). Fat is distributed gently above the dorsal deep fascia to avoid perforation of the vessels. Antibiotic prophylaxis is always provided with cephalexin 1 g. every 12 h for 7 days. Posttreatment check-ups are performed at 24 h, 7 days, 1 month, 3 months, and 12 months after treatment to evaluate the healing of the hand (Fig. 7).

## Results

**Fig. 8:**
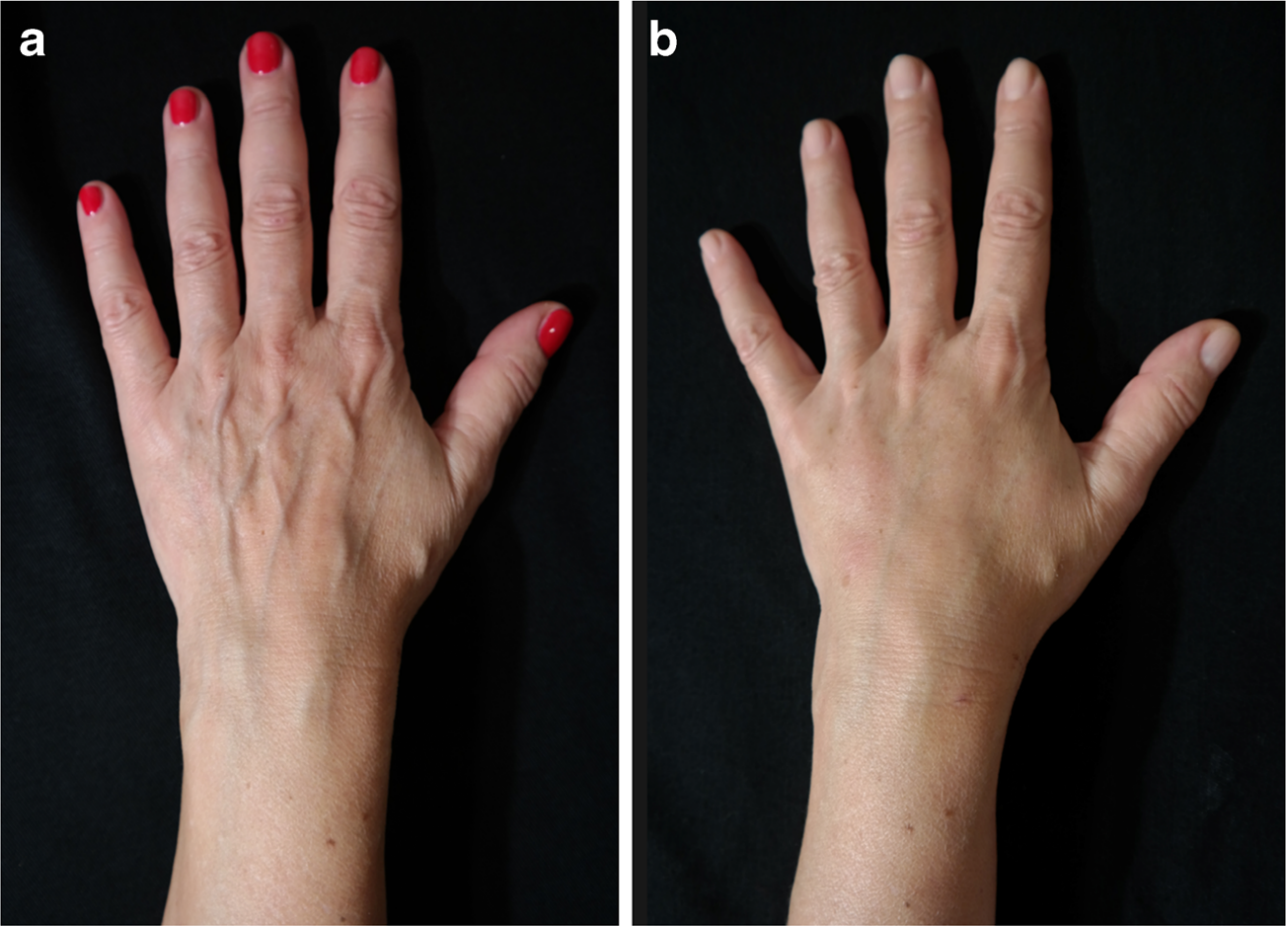
A 41-year-old female patient (left hand): **a** preoperative and **b** postoperative view at 9-months

**Fig. 9:**
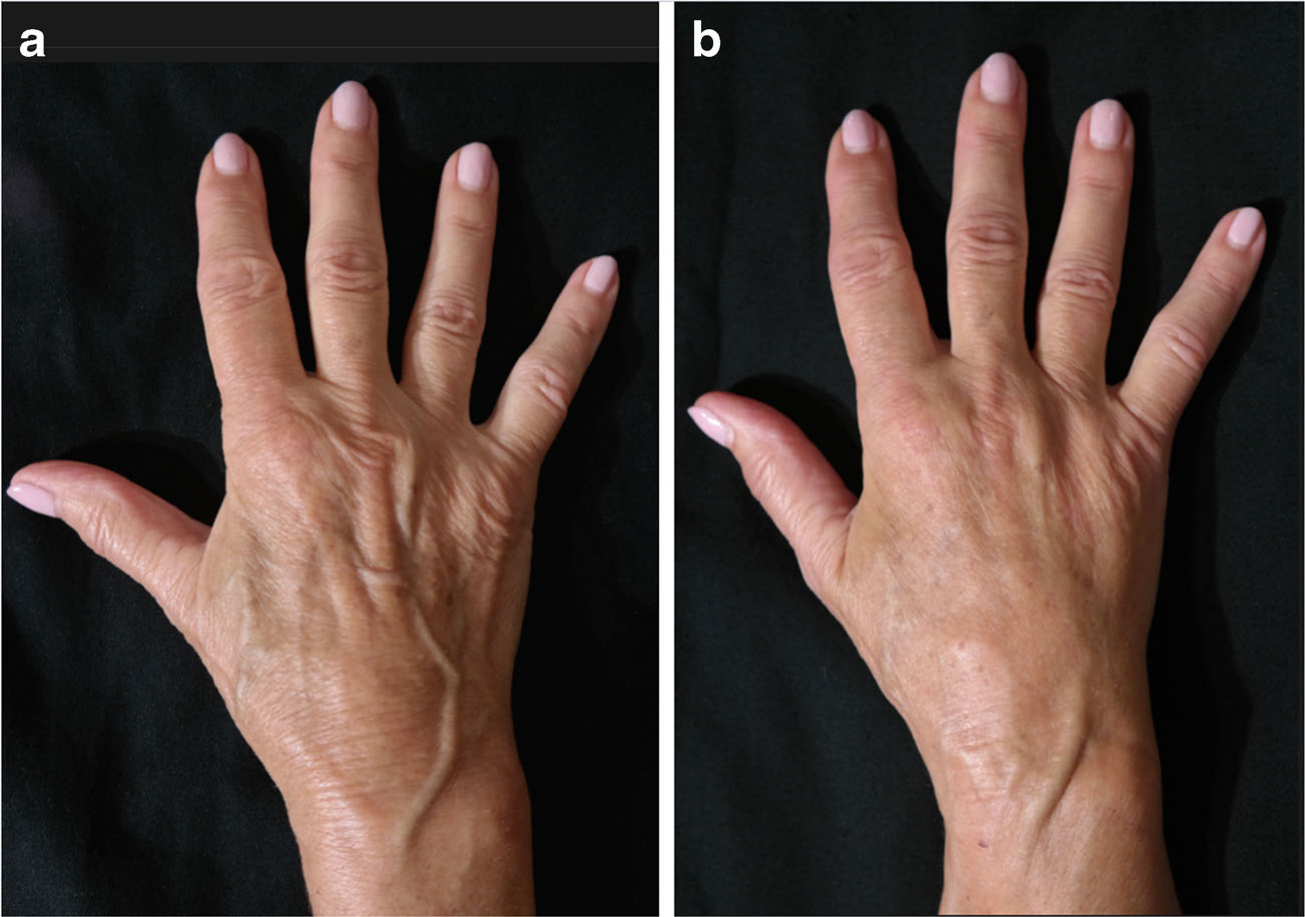
A 80-year-old female patient (right hand): **a** preoperative and **b** postoperative view at 1 year

**Fig. 10:**
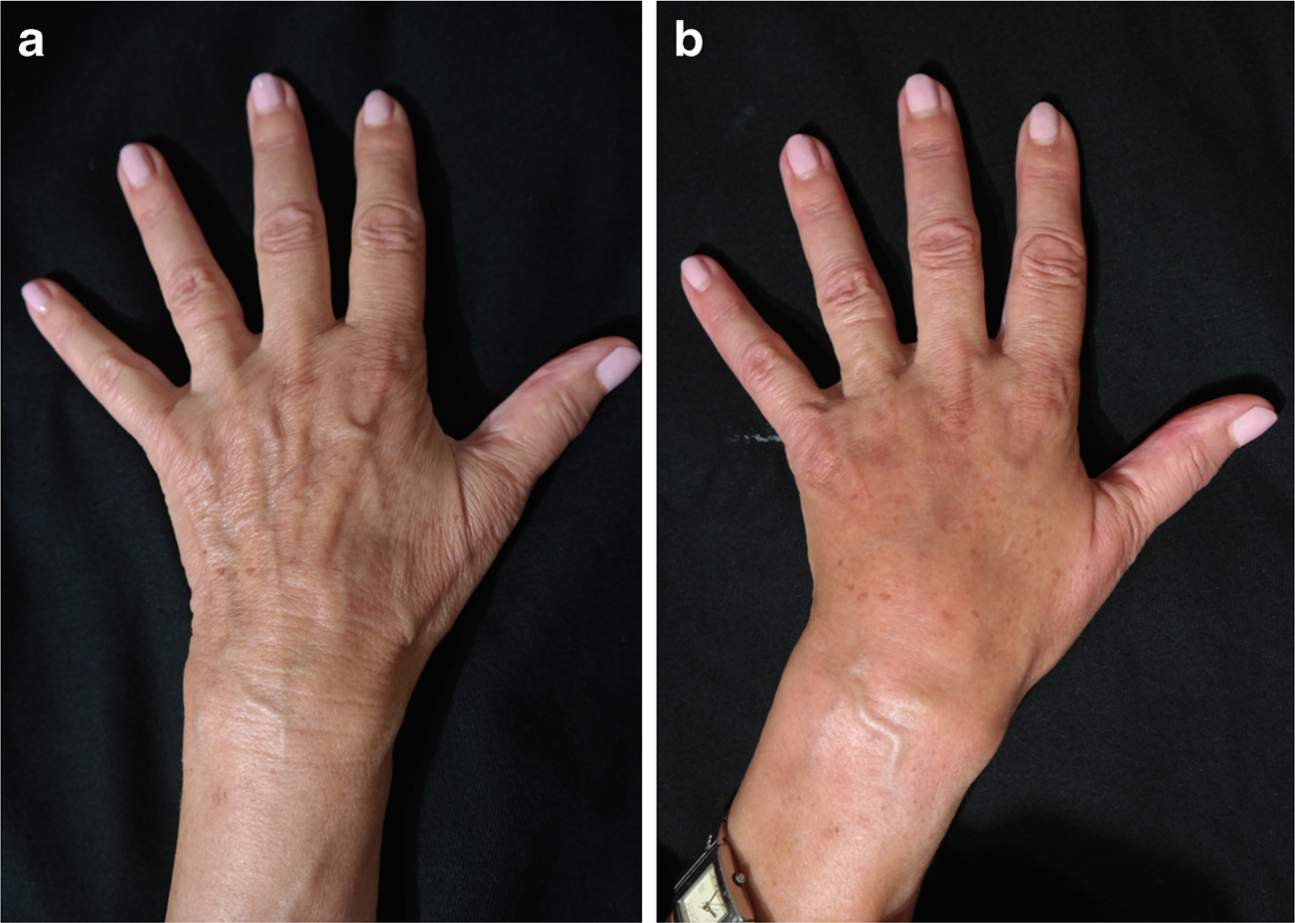
A 80-year-old female patient (left hand): **a** preoperative and **b** postoperative view at 1 year

**Fig. 11:**
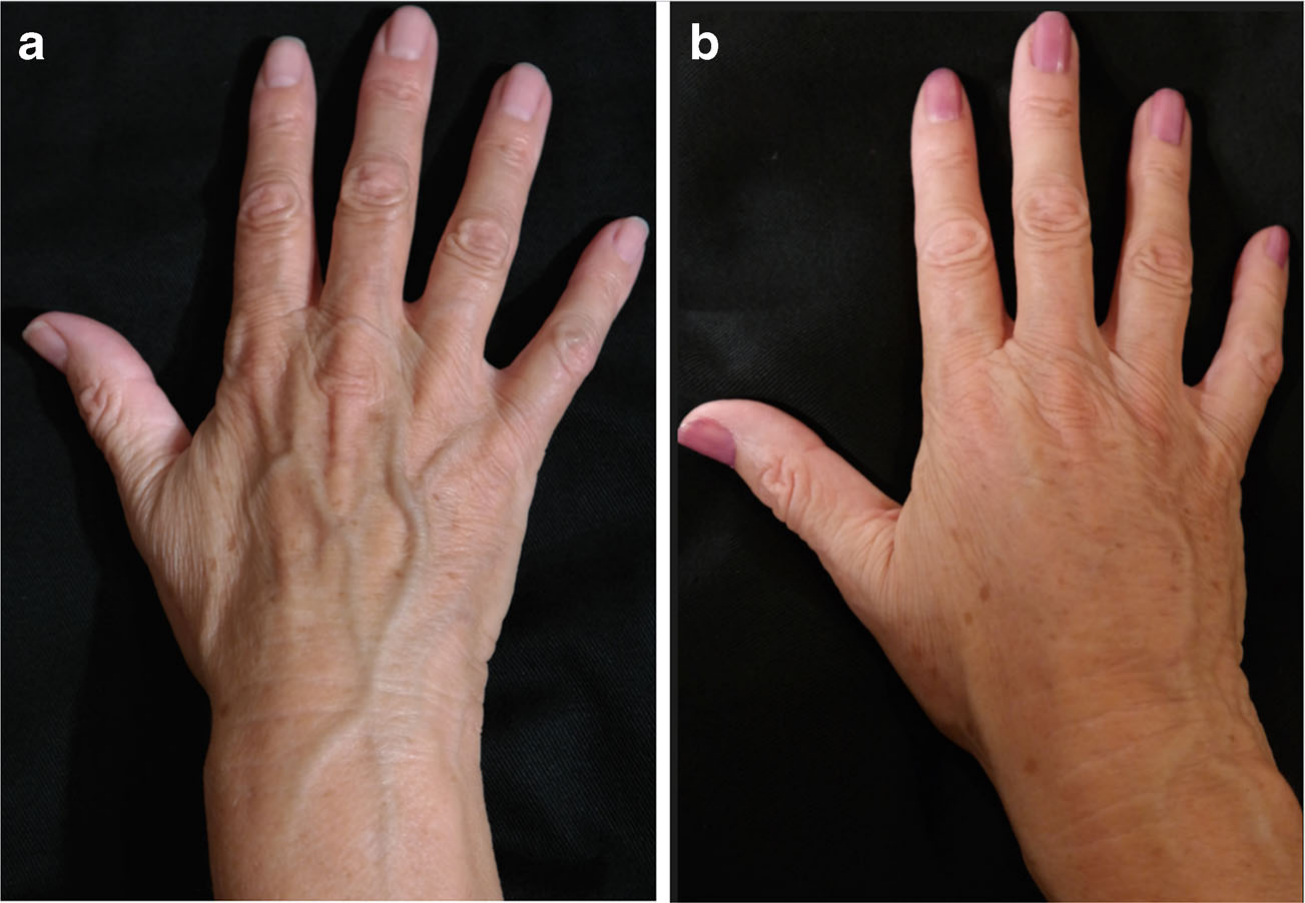
A 72-year-old female patient (right hand): **a** preoperative and **b** postoperative view at 10 months

**Fig. 12:**
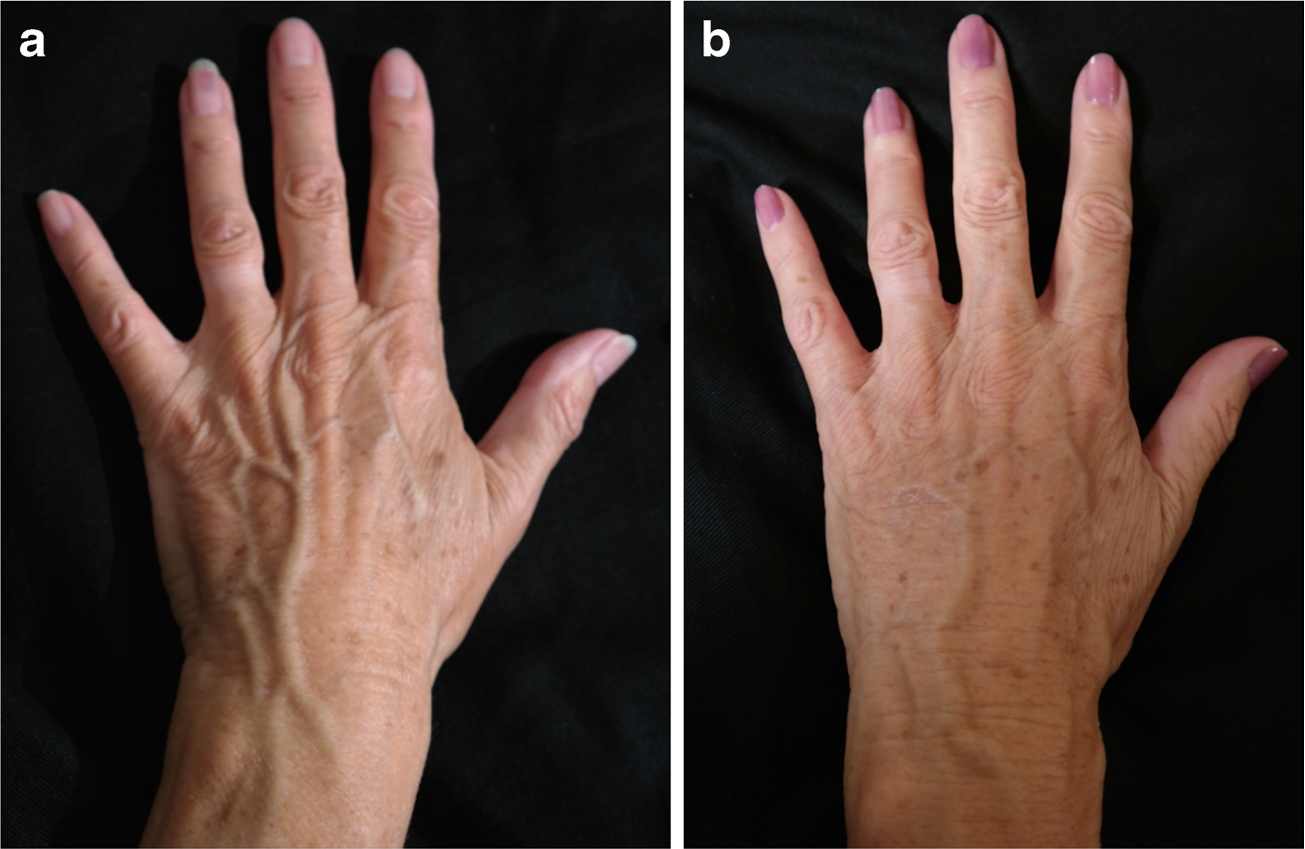
A 72-year-old female patient (left hand): **a** preoperative and **b** postoperative view at 10-months

**Fig. 13:**
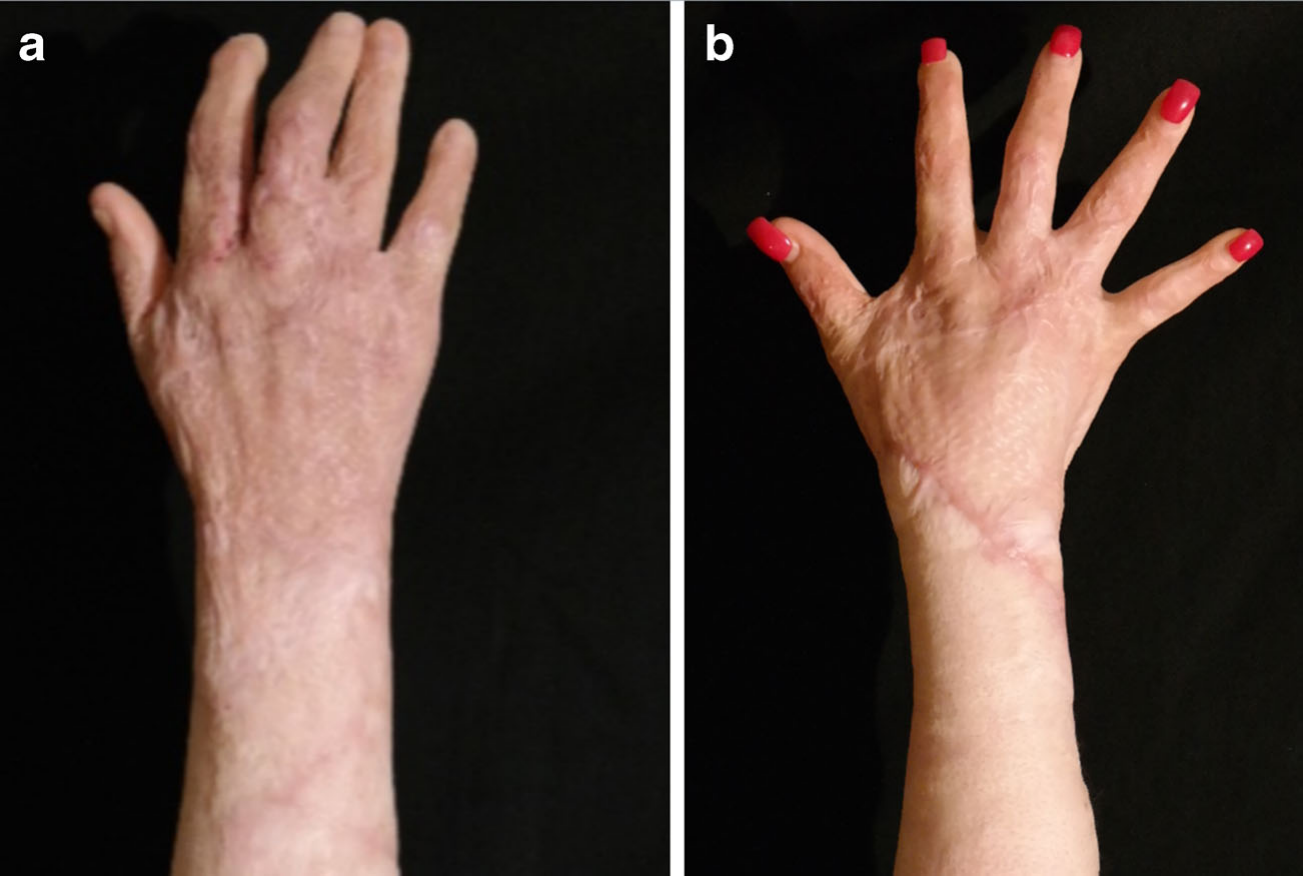
A 22-year-old female patient who suffered from burn injury to her right hand: **a** preoperative and **b** postoperative view at 1 year after three rounds of lipofilling and 30 mL of transferred fat in total

Sixty-five patients underwent the hand fat grafting procedure and were included in this study. Each patient’s progress was followed-up for a minimum of 12 months during personal consultation. Over this period, contour changes in their hands and the effects of the procedure(s) on their skin were analyzed. Fifty-six patients (84%) were satisfied with the results during the observation period; 7 patients (12%) were somewhat satisfied and needed one more fat grafting procedure to achieve complete satisfaction, and of these, 6 were smokers, 3 had hyperthyroidism, 1 was a user of diet fat burning pills, and 1 was a burns patient. Two patients (4%) were dissatisfied with the results. Three cases of temporary swelling of the hands resolved spontaneously. No long-term complications were seen. Tables 1 and 2 show the patients’ demographics and postoperative complications, respectively. Representative cases are depicted in Figs. 8, 9, 10, 11, 12, and 13.

**Table 1:** Patients’ demographics

Case studies—statistical	
Number of patients operated upon	65
Average age of patients	51.3 years
Youngest patient	33 years
Oldest patient	81 years
No. of female patients	50/65
No. of male patients	15/65
Average time required for procedure	20 min

**Table 2:** Postoperative complications

Post-op complications	
Permanent/long-term complications	None
Temporary paresthesia	7/65 patients (resolved naturally within 48 h)
Temporary swelling of hands	3/65 patients (resolved naturally within 72 h)
Patients with follow-up period of at least 12 months	65

## Discussion

The developed procedure is aimed at treating signs of aging in the hand. The appearance of the hands changes significantly during our lives. The five esthetic characteristics of young people’s hands change with age owing to both intrinsic factors (epidermal and dermal change) and extrinsic factors (deeper change) [5]. The proposed procedure treats intrinsic factors such as the loss of subcutaneous fat and the visibility of veins and tendons. A dermatologist from our team treats extrinsic factors such as alteration in cutaneous pigmentation.

We create new, fat-cell-rich subcutaneous tissue, resulting in noticeable skin rejuvenation. The large number of fat cells has beneficial effects on deep and superficial skin tissue. A substantial amount of fat tissue on the dorso results in greatly reduced skin flaccidity and barely visible superficial veins. The application of our treatment results in better skin quality, thereby rejuvenating the hand. Giunta et al. also reported excellent hand restoration results after lipofilling [6].

The lipofilling procedure affords many advantages. It restores subcutaneous fat loss in the hand, thereby covering visible veins and tendons and reducing skin flaccidity. The long-term effect of fat grafting is hand rejuvenation, as a higher percentage of fat cells in the subcutaneous region have beneficial effects such as dermal regeneration [5–8].

Many studies have reported adverse reactions when using synthetic fillers [9]. However, fat cells represent a biologic filler that can be safely used by a plastic surgeon for tissue filling [1, 2]. No allergic reactions occur in our series, because the patient’s own fat cells are used.

Coleman’s approach for lipofilling treats fat by centrifugation [10]; instead, our proposed approach treats fat by decantation before fat grafting. No centrifuge machine is used to prevent fat damage [11, 12]. As also reported by Lee [12], treating fat by decantation alone inside a syringe is safe and shows good results. Furthermore, this procedure is fast and safe for lipofilling. In fact, some studies have expressed doubts regarding fat cell centrifugation and noted that it could lead to some deterioration of the fat tissue [13, 14]. On the other hand, grafting is performed using a cannula from the wrist and not from the fingers. Our proposed approach shares some aspects with Fournier’s technique, such as the use of a syringe to obtain the fat, same cannula for fat injection, and average volume of fat injected [15].

Autologous fat grafting was found to be a useful method because it showed long-lasting results after a 1-year follow-up. It was necessary to repeat the process to improve the result only in some isolated cases.

Hand fat grafting can even be combined with a light peel and electrocoagulation to treat senile keratosis. Abergel noted that laser resurfacing after lipofilling is effective for treating the alteration of cutaneous pigmentation [16]. In fact, we recommend laser treatment after fat grafting for those patients with actinic keratosis and solar lentigines.

Some studies have reported complications such as infection after hand lipofilling [17, 18]. This is the only complication that typically needs to be prevented in this procedure. It can be avoided by ensuring that sterility is maintained. Sterile or disposable cannulas should be used to extract and inject fat. Furthermore, antibiotics should be always prescribed for 1 week as a precaution. Infection of the hands is a rare, but extremely dangerous, complication. In particular, if infection is not diagnosed in time in elderly patients, hospital admission may be required for treatment [18].

## Conclusion

This study covers over a decade of practical experience in applying fat grafts to the hands. Autologous fat grafting is effective as it shows long-lasting results after 1-year follow-up. Hand fat grafting is an addition to the armamentarium of plastic surgeons for reshaping and rejuvenation of the hands.
